# Welfare impacts of improved chickpea adoption: A pathway for rural development in Ethiopia?

**DOI:** 10.1016/j.foodpol.2016.11.007

**Published:** 2017-01

**Authors:** Simone Verkaart, Bernard G. Munyua, Kai Mausch, Jeffrey D. Michler

**Affiliations:** a International Crops Research Institute for the Semi-Arid Tropics (ICRISAT), Kenya; b Development Economics Group, Wageningen University & Research, The Netherlands; c Department of Agricultural and Consumer Economics, University of Illinois, United States; d International Crops Research Institute for the Semi-Arid Tropics (ICRISAT), Zimbabwe

**Keywords:** Improved chickpea, Technology adoption, Poverty, Control function, Ethiopia

## Abstract

We analyse the impact of improved chickpea adoption on welfare in Ethiopia using three rounds of panel data. First, we estimate the determinants of improved chickpea adoption using a double hurdle model. We apply a control function approach with correlated random effects to control for possible endogeneity resulting from access to improved seed and technology transfer activities. To instrument for these variables we develop novel distance weighted measures of a household’s neighbours’ access to improved seed and technology transfer activities. Second, we estimate the impact of area under improved chickpea cultivation on household income and poverty. We apply a fixed effects instrumental variables approach where we use the predicted area under cultivation from the double hurdle model as an instrument for observed area under cultivation. We find that improved chickpea adoption significantly increases household income while also reducing household poverty. Finally, we disaggregate results by landholding to explore whether the impact of adoption has heterogeneous effects. Adoption favoured all but the largest landholders, for who the new technology did not have a significant impact on income. Overall, increasing access to improved chickpea appears a promising pathway for rural development in Ethiopia’s chickpea growing regions.

## Highlights

• We study the impact of improved chickpea adoption using three rounds of panel data.
• Adoption is instrumented using a control function approach and double hurdle model.
• Improved chickpea increased household income and reduced $2.00 (median) poverty.
• We find strong impacts for the three lower landholding quartiles.
• Improved chickpea are a promising pathway for rural development in Ethiopia.

## 1 Introduction

Ethiopia is among the poorest countries in the world, is highly drought-prone and has an agricultural sector that accounts for 85 percent of employment (Dercon et al., 2012, Spielman et al., 2010). Exacerbating the situation, Ethiopia’s population of 92 million is expected to grow to 160 million by 2050 (Josephson et al., 2014). As a result, farm sizes have been rapidly declining, increasing the need for agricultural intensification (Headey et al., 2014). Accordingly, increasing the productivity of smallholders through improved technology has become a policy priority for development agencies as well as the Ethiopian government (Abebaw and Haile, 2013). It has been suggested that tropical legumes can contribute to poverty reduction by improving food security and incomes of smallholder farmers in Africa (Gwata, 2010). One particularly promising technology is high yielding, drought tolerant chickpea varieties which can be used for on-farm consumption as well as export.

In this paper, we analyse the impact of adopting improved chickpea varieties on household welfare in rural Ethiopia. To do so we employ three rounds of panel data (2006/07, 2009/10, 2013/14) with a control function approach and instrumental variable estimation to control for endogeneity of access to improved seed, technology transfer activities and adoption.^1^ We seek to answer the following research questions: What has been the impact of improved chickpea adoption on household income? To what extent did adoption contribute to poverty reduction? And, did adoption affect households differently depending on initial wealth status?

To motivate our empirical analysis, we first develop a simple conceptual framework using a non-separable model of a farm household that is simultaneously involved in both production and consumption decisions. We then estimate the area under improved chickpea using a double hurdle model. We apply a control function approach with correlated random effects to control for possible endogeneity of access to improved seed and participation in chickpea technology transfer activities. We develop a novel distance weighted measure to instrument for these endogenous regressors. Finally, we estimate the impact of area under improved chickpea on household income and poverty. We apply a fixed effects instrumental variables model where we use the predicted values from the double hurdle model as an instrument for observed area under improved chickpea cultivation.

Our primary contribution is to provide rigorous evidence on the impact of agricultural technology adoption on household income and poverty reduction. This comes at a particularly relevant time, as the 68th United Nations General Assembly declared 2016 the International Year of Pulses. Improved chickpea adoption increased dramatically from 30 to almost 80 percent of the sampled households between the 2006/07 and 2013/14 seasons. We find that adoption has a positive and significant impact on household income. Furthermore, households that adopt improved chickpea are less likely to be poor than households that choose not to adopt. We also isolate the impact of improved chickpea adoption on income based on a household’s initial land ownership. Improved chickpea adoption has a positive and significant impact on income for households with landholding in the three lower quartiles, but no significant effect on the income of the largest landholding households. The beneficial biotic and nutritional characteristics of legumes combined with our positive findings, implies that there is considerable potential for upscaling improved chickpea distribution networks for rural development in Ethiopia.

Our research contributes to a growing literature on the impact of technology adoption on poverty and income in Sub-Saharan Africa, which has been thin and mixed (Cunguara and Darnhofer, 2011, Kassie et al., 2011). Much of the previous work has focused on hybrid maize, either in Kenya (Mathenge et al., 2014), Malawi (Bezu et al., 2014) or Zambia (Mason and Smale, 2013, Smale and Mason, 2014). Previous research on the impact of improved varieties of legumes does exist but, to date, has been hampered by data limitations. Research on chickpea in Ethiopia (Asfaw et al., 2012, Asfaw et al., 2010), groundnut in Malawi (Simtowe et al., 2012) and groundnut in Uganda (Kassie et al., 2011) all relied on cross-sectional data, which limited the ability of these studies to identify causal impacts. To our knowledge, no research exists that identifies the impact of improved legume adoption on farmer welfare in Sub-Saharan Africa.

## 2 Background: chickpea production in Ethiopia

Chickpea is an important crop in Ethiopia. The country is the seventh largest producer in the world and accounts for over 90 percent of Sub-Saharan Africa’s chickpea production (Kassie et al., 2009, Pachico, 2014). In Ethiopia chickpea is grown in rotation with cereals (primarily teff and wheat) and does not directly compete for land and labour with these cereals. Kassie et al. (2009) suggested that improved chickpea varieties are a key pro-poor and environmentally friendly technology for agricultural development and economic growth in Ethiopia. First, the growing demand in both the domestic and export markets provides a source of cash for smallholder producers (Abera, 2010, Shiferaw and Teklewold, 2007). Second, chickpea are considered environmentally friendly due to their capacity to fix atmospheric nitrogen and reduce chemical fertilizer use and costs in subsequent cereal crops (Giller, 2001). Finally, chickpea and its residues are a source of protein and can reduce malnutrition (Malunga et al., 2014, Sarker et al., 2014) and/or increase livestock productivity (Macharia et al., 2012).

The ability of Ethiopia’s chickpea sector to foster economic growth and development depends on the country’s ability to improve productivity, enhance grain quality and consistently supply the required volumes of market-preferred products at competitive prices (Abera, 2010, Keneni et al., 2011). More than ten improved chickpea varieties have been released (Asfaw et al., 2012). But until 2004, insufficient seed production limited the availability of quality seeds and the adoption of improved varieties was low (Shiferaw et al., 2007). Various initiatives were started to accelerate the adoption of improved chickpea varieties in Ethiopia. The Ethiopian Institute of Agricultural Research (EIAR) cultivated partnerships with major actors along the value chain to support the adoption of improved varieties (Abate et al., 2011). Primary co-operatives received breeder seed and multiplied them using contract farmers to enable the dissemination of improved chickpea varieties (Shiferaw et al., 2007). Moreover, the Tropical Legumes II (TLII) development program has conducted various chickpea research and development activities, including the establishment of seed grower associations (Monyo and Varshney, 2016).^2^ TLII focused on major chickpea producing areas in the Shewa region for the upscaling of suitable chickpea varieties and marketing strategies. Other developments that boosted the chickpea sector included the decision to include chickpea in the Ethiopian commodity exchange and formation of the multi-stakeholder EthioPEA alliance.

Access to improved seeds and chickpea technology transfer are important pre-conditions for adoption. Krishnan and Patnam (2013) suggested that technology transfer activities provided by extension agents in Ethiopia transmit information vital to farmers in the early stages of adoption. They also found, however, that learning from neighbours who have adopted is more important than extension for the further diffusion of technologies. On chickpea, Asfaw et al. (2012) found that relatively affluent farmers had better access to improved seed in our study area which suggests that richer farmers might have been targeted through the extension system. They further note that Lume-Ejere district (one of our study areas) is strategically located on a main interstate road and closest to the national research centre that developed improved chickpea varieties, which might have disproportionately benefited farmers in the district in the form of pre-extension demonstrations and improved seed distribution trials. This suggests that access to improved variety seed and chickpea technology transfer activities in the area was neither universal nor random. We adopt an instrumental variables approach to address the non-random access to improved varieties.

## 3 Conceptual framework

It is too simplistic to assume that promoting agricultural technologies will automatically boost productivity, improve livelihoods and alleviate poverty (Tittonell, 2007). The potential effect of technology transfer depends on whether farmers adopt and, if they do, whether they adopt the technologies in an ideal combination and for the prescribed length of time needed to produce results (Parvan, 2011). For innovations that are ‘divisible’ and can be adopted in a stepwise manner the adoption decision involves a choice regarding the intensity of adoption (Marra et al., 2003). Adoption decisions are generally assumed to be the outcome of optimizing expected profit, where returns are a function of land allocation, the production function of the technology and the costs of inputs and prices of outputs (Feder et al., 1985). Often cited factors used to explain adoption are farm size, risk, human capital, labour availability, credit constraints, land tenure and access to input and output markets (Feder et al., 1985, Foster and Rosenzweig, 2010, Sunding and Zilberman, 2001). Adoption choices are also conditioned on agro-ecological characteristics, such as soil quality, rainfall patterns and the farming system (Mason and Smale, 2013). Adoption of improved varieties also depends on the availability and accessibility of improved seeds and training in chickpea cultivation (Asfaw et al., 2012), which is a concern in our context.

Further complicating measurement of adoption and its impact on welfare is the non-separability of household production and consumption decisions. In Ethiopia, smallholder farmers operate in an institutional environment characterized by failures in the labour, input and credit markets (Asfaw et al., 2012, Gebremedhin et al., 2009, Teklewold et al., 2013). As a result, households are simultaneously involved in both production and consumption decisions and the assumption of separability between these decisions is unlikely to hold. Accordingly, we analyse improved chickpea adoption using a non-separable model of the farm household, in which family members organize their labour to maximize utility over consumption goods and leisure in an economic environment with market failures (de Janvry et al., 1991).

Households produce goods for consumption or sale and cash constraints are relaxed primarily through farm sales of surplus products and off-farm income (Smale and Mason, 2014). Household endowments of natural, human, financial, physical and social capital constitute the resource constraints based on which well-being is maximized. In addition to factors of production, our model of adoption includes household demographic characteristics. Let K represent the area of land planted with improved chickpea:

(1) K=f(X,L,T,Z,V)

where X is a household’s ability to cultivate improved seed (which incorporates both access to improved seed and technology transfer), L is the household’s labour endowment and T is household demographic characteristics. Additional determinants include agro-ecological characteristics (Z) and village level covariates (V).

It is important to estimate the impact of technology adoption on household income and poverty, because this gives a measure of the extent to which the technology actually affects household welfare (de Janvry et al., 2011). Here we consider household welfare in a utility framework such that

(2) Y=f(K,L,T,V)

where Y is household welfare and other variables are as previously defined. We use the two stage approach given in Eqs. (1), (2) to guide our empirical estimation procedure.

While conceptually both household welfare and technology adoption are functions of labour endowment, household demographic characteristics and village level covariates, the specific variables included need not have the same effect in both functions. As an example, the amount of off-farm income a household earns is likely to decrease adoption of improved chickpea (as farming is relatively less important) while it is likely to increase household welfare. With respect to our primary variable of interest, we hypothesize that growing higher yielding improved varieties will increase household income. This positive impact on welfare may be direct, through selling surplus chickpeas, or indirect, by releasing land to produce other crops for sale. If farmers use improved varieties successfully over several seasons, we expect that incremental increases in income could be capitalized to raise households above the poverty threshold (Mathenge et al., 2014). Accordingly, we test for a positive and significant impact of adoption of improved chickpea on both household income and poverty status. However, in contexts where households hold large areas of land on which they grow a wide diversity of crops or have other income sources, the average impact of adopting the improved variety could be insignificant (Mason and Smale, 2013). Therefore, in subsequent analysis, we allow for chickpea adoption to have heterogeneous effect on income depending on a household’s initial level of land ownership.

## 4 Data and descriptive statistics

### 4.1 Data

Our data comes from major chickpea producing areas in the Shewa region. Shewa is northeast of Debre Zeit, which is 50 km southeast of Addis Ababa. From the regions that have a suitable agro-ecology for chickpea production, three districts (Minjar-Shenkora, Gimbichu and Lume-Ejere) were purposely selected based on the intensity of chickpea production. In each district, eight to ten villages were randomly selected and within these 150–300 households were randomly selected. A total of 700 farm households in the three districts were surveyed using a standardized survey instrument. Accordingly, our results are not nationally representative and should be interpreted as an upper bound of the potential impacts of improved chickpea adoption in the whole of Ethiopia.^3^ The districts are in the central highlands at an altitude ranging from 1700 to 2700 meters. Chickpea is grown during the post-rainy season on black soils using residual moisture. Debre Zeit Agricultural Research Centre (DZARC) is located in the area and is a source of information and improved crop varieties, including chickpea.

We analyse the impact of improved chickpea variety adoption on household welfare in Ethiopia using three rounds of panel data (2006/07, 2009/10 and 2013/14). During the three survey rounds 700, 661 and 631 households were surveyed respectively. Since households were randomly selected both chickpea and non-chickpea growers were interviewed. Our analysis utilizes a balanced sample of 606 households. Balancing the panel results in an attrition rate of 13 percent. To check for non-random attrition we compared characteristics using the first round of data collected and found no significant differences between attritors and non-attritors.^4^

To enable comparisons across time, we deflated nominal Ethiopian Birr values to real values using the national consumer price index with 2005 as a base. These constant 2005 Ethiopian Birr data were subsequently converted to US dollar (USD) Purchasing Power Parity (PPP) values using rates extrapolated from the 2011 International Comparison Program (World Bank, 2015b).^5^ We consider both the international poverty line of 1.25 USD PPP and median poverty line of 2.00 USD PPP per day per capita (both in constant 2005 prices), which represent the lower and upper bounds of poverty (Ravallion et al., 2009). We calculate household welfare as annual net income per capita in constant 2005 USD PPP. We explicitly account for input and hired labour costs for crop production and livestock rearing using detailed information in our data regarding farm production.

Adopters are defined as households who plant an improved chickpea variety. As our measure of adoption in the econometric models we use the area allocated to improved varieties as an indicator for the extent or scale of adoption. Misidentification of varietal types is a common problem in many studies of adoption. This has led to a much more rigorous approach, sometimes using DNA fingerprinting, as a way to verify that farmers are actually growing what they say they are growing. However, the improved varieties in this study are predominantly newly introduced Kabuli chickpea types (95% of improved varieties). Kabuli were not traditionally cultivated in Ethiopia and are easy to distinguish from traditional Desi varieties. Kabuli are larger and cream coloured while Desi are smaller and brown. Additionally, the two varieties have a different flower colour. We are therefore confident that improved seed is correctly identified.

### 4.2 Descriptive statistics

Adoption of improved chickpea increased dramatically from 30 to almost 80 percent of the total sample over the study period (Table 1). In line with adoption, seed and land allocated to improved chickpea increased. Chickpea growers allocated half a hectare to improved varieties and it contributed up to twenty percent of total household income.

Table 2 indicates that there are systematic differences between adopters and non-adopters. Adopter households had significantly larger families in the first two rounds. Other demographic indicators, including the head of the household’s gender, education and age, did not differ between the two groups, though first round adopters had better educated household heads. Adopters were considerably wealthier than non-adopters, with higher total and per capita incomes across all three rounds. Differences in income and land become less stark over time, suggesting that early adopters were notably wealthier. Finally, poverty rates were substantially lower among adopters across the three rounds.

**Fig. 1:**
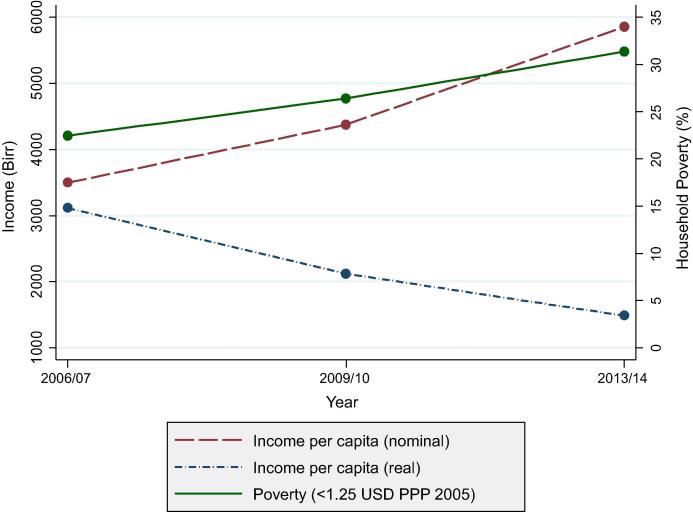
Poverty trends and income per capita in real and nominal Ethiopian birr.

In this study we are interested in the dynamics of poverty, in particular, how poverty status changes with the adoption of improved chickpea. Though nominal incomes increased considerably between 2006/07 and 2013/14, real incomes could not keep up with the high inflation experienced in Ethiopia (Fig. 1). In 2011, Ethiopian food inflation was 39 percent, three times the Sub-Saharan African average of 13 percent (World Bank, 2015a). As a result, poverty increased from 22 to 31 percent over the study period.

**Fig. 2:**
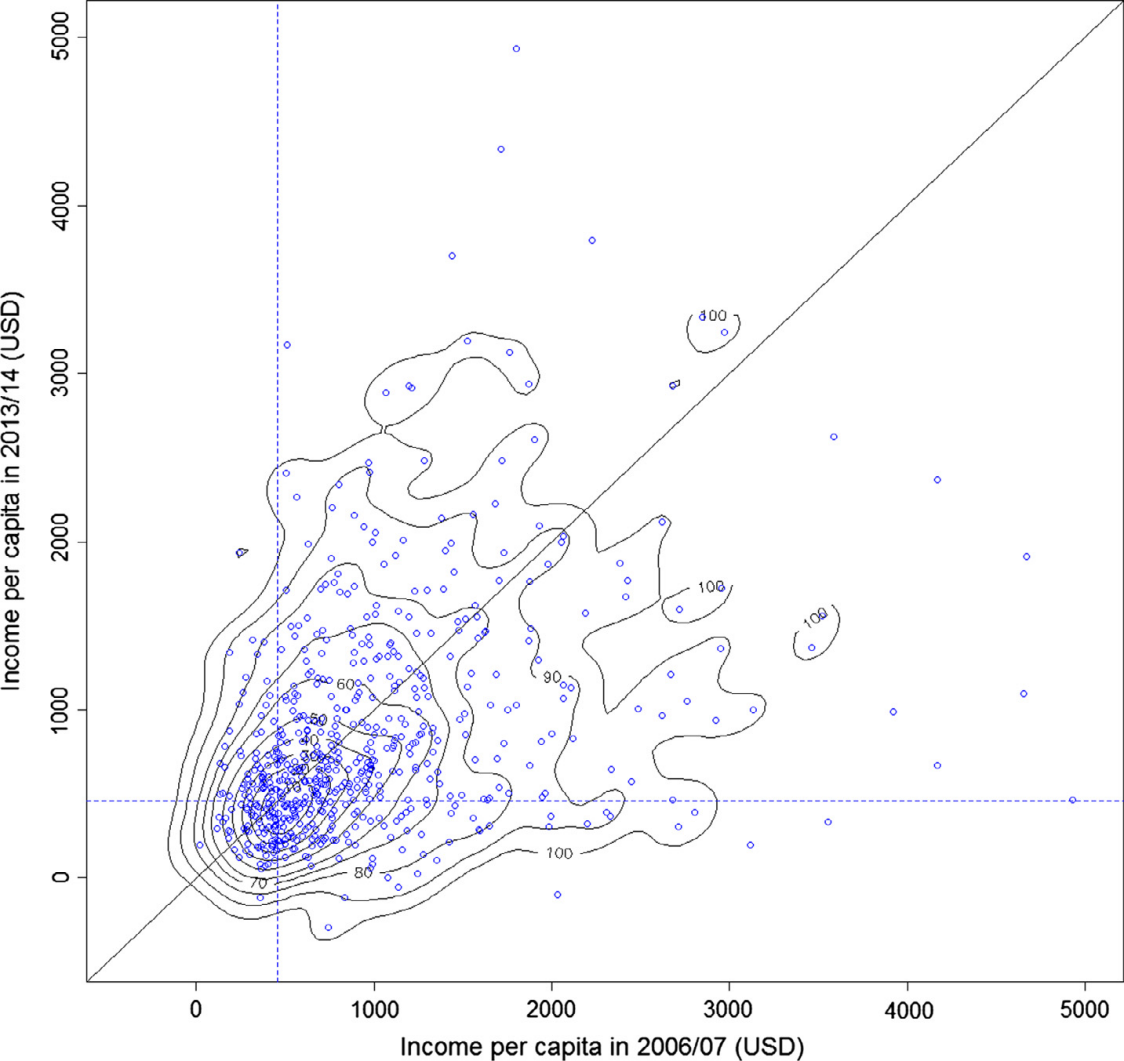
Bivariate density of mean real income per capita (constant 2005 USD PPP).

To better understand how household poverty changed over time we use data from 2006/07 and 2013/14 to draw the bivariate kernel density contours of real income per capita in constant 2005 USD PPP (see Fig. 2). Circles indicate observed household data. To this, we have added dashed lines indicating the poverty line of 1.25 per day (constant 2005 USD PPP) and a solid 45° line. Households above the 45° line have more per capita income in 2013/14 than in 2006/07. Households below the 45° line have less per capita income in 2013/14 than in 2006/07. As expected, most of the mass lies below the 45° line with 57 percent of households having less real per capita income in 2013/14 than in 2006/07.

Despite this loss in real per capita income, most households remained above the $1.25 poverty line. In fact, 59 percent of households were above the poverty line in 2006/07 and remained above the poverty line in 2013/14 (these households are in the northeast quadrant of Fig. 2). A significant share of households, 19 percent, were above the poverty line in 2006/07 but by 2013/14 had fallen into poverty (southeast quadrant). Twelve percent of households started the study period in poverty and saw no change in their fortunes (southwest quadrant). Only 10 percent of households began 2006/07 below the poverty and were able to rise out of poverty by 2013/14 (northwest quadrant).

## 5 Empirical approach

### 5.1 Estimation of improved chickpea adoption

The objective of this study is to analyse the impact of improved chickpea adoption on household welfare. Starting from Eq. (1) in our conceptual model we specify the following

(3) $${\text{K}}_{\mathrm{it}}=\alpha +\beta_{1}X_{\mathrm{it}}^{\mathrm{TT}}+\beta_{2}X_{\mathrm{it}}^{\mathrm{IS}}+T_{\mathrm{it}}\theta +Z_{i}\zeta +D_{t}+v+{∊}_{\mathrm{it}}$$

where ${\text{K}}_{\mathrm{it}}$ is the area planted with improved chickpea by household i in year t and $X_{\mathrm{it}}^{\mathrm{TT}}$ and $X_{\mathrm{it}}^{\mathrm{IS}}$ are our measures of access to technology transfer and improved chickpea seed, respectively. $T_{\mathrm{it}}$ is a vector of household characteristics and $Z_{i}$ is a vector of time-invariant agro-ecological characteristics both of which influence the desirability of adopting improved chickpea. We also include year, $D_{t}$, and village, v, dummies to control for common shocks and unobserved regional characteristics that affect improved chickpea adoption. Finally, ${∊}_{\mathrm{it}}$ is a compound error term consisting of unobserved time-invariant factors, $c_{i}$, and unobserved time-variant shocks, $\upsilon_{\mathrm{it}}\text{,}$ that affect improved chickpea adoption.

Table 3 provides descriptive statistics for the variables used in the model. Estimation of Eq. (3) is complicated by several econometric issues which make causal identification difficult. We address these in turn.

#### 5.1.1 Unobserved heterogeneity

A first estimation issue is the presence of household heterogeneity that influences adoption but is otherwise unobserved. This unobserved heterogeneity creates selection bias as some households are more likely to adopt improved chickpea varieties than other households. The standard panel data method would be to include household fixed effects, which allow for arbitrary correlation between $c_{i}$ and our household variables. However, the prevalence of households that grow no improved chickpea means that the data takes on properties of a non-linear corner solution (Ricker-Gilbert et al., 2011). To avoid the incidental variables problem that fixed effects introduce in non-linear models we adopt a correlated random effects framework, first pioneered by Mundlak, 1978, Chamberlain, 1984. We assume that the unobserved heterogeneity can be replaced with its linear projection onto the time averages of all exogenous variables such that

(4) $$c_{i}={\overset{‾}{T}}_{i}\lambda_{1}+u_{i}\text{.}$$

While not as weak of an assumption as used in fixed effects, since we specify the correlation between $c_{i}$ and our household variables, correlated random effects does relax the strong assumption of no correlation required in a random effects model (Wooldridge, 2010).

#### 5.1.2 Unobserved shocks

A second estimation issue is the possible presence of unobserved shocks captured in $\upsilon_{\mathrm{it}}$ that might affect a household’s access to and cultivation of improved chickpea. Given that the improved chickpea seed system is in its infancy, farmer’s access to seed during the period of study was limited (Abate et al., 2011). Few farmers bought chickpea seeds and the percentage of farmers buying seed reduced over time, suggesting that seed replenishment rates went down. However, some farmers could access improved seed through buying (from the market), borrowing (from a revolving seed fund) or receiving as a gift (from friend/family/neighbour). In addition, activities designed to improve farmer capacity were not universally available. Technology transfer activities include farm trials or demonstrations, farmer field days, farmer training centres, field schools and seminars, and participation in these activities increased over time. Seed dissemination and extension activities were often targeted to specific villages and farmers (Asfaw et al., 2012, Shiferaw et al., 2007). This means that access to technology transfer and improved seed are neither random nor static and thus likely correlated with unobserved time-varying factors.

To control for unobserved shocks we adopt an instrumental variables approach. An appropriate instrument for this model must be correlated with a household’s access to technology transfer and improved chickpea seed but uncorrelated with the amount of land under improved chickpea cultivation. We develop a spatial measure of access to improved seed and another for participation in technology transfer, each of which excludes a farmer’s own access to seed and participation in technology transfer. The idea is that if neighbouring households have access to improved seed (participated in technology transfer), this will translate into a higher probability that the farmer in question will have access to improved seed (technology transfer). To ensure that causality does not run in reverse (farmer to neighbour instead of neighbour to farmer), we use the lagged value of each of our spatial measures as the instruments.

To construct our instruments we incorporate insights from recent research on the importance of social networks in technology adoption (Conley and Udry, 2010, Krishnan and Patnam, 2013, Magnan et al., 2015). While our data does not include information of social interactions or networks, it does include GPS coordinates for all households. We use this information to measure the distance between each surveyed household in a village. We also measure the distance between each household and every other surveyed household in the village that had access to improved chickpea seed (technology transfer). Using the inverse of these distances so that higher values correspond to nearer neighbours, we calculate two distance weighted ratios (one for access to seed and one for technology transfer) of neighbours with access to improved seed (technology transfer) to all households surveyed in the village. Thus,

(5) $$W_{\mathrm{it}}=\left(\sum \frac{x_{\mathrm{jt}}}{d_{\mathrm{ij}}}\right)/\left(\sum \frac{1}{d_{\mathrm{ij}}}\right))$$

where $W_{\mathrm{it}}$ is the distance weighted ratio at time t of those with access to improved seed (technology transfer), $x_{\mathrm{jt}}$ is an indicator equal to one if neighbour j had access to improved seed (technology transfer) at time t and zero otherwise and $d_{\mathrm{ij}}$ is the distance between household i and household j. While distance is time-invariant, access to improved seed (technology transfer) varies from year to year so that our instrument is time-variant. By using distance to weight the binary variable indicating if a household had access to improved seed (technology transfer), we incorporate the idea that nearby households are more likely to be part of the same social network. Thus, a nearby household with access to improved seed (technology transfer) will have a larger impact on $W_{\mathrm{it}}$ than a distant household’s access. By expressing $W_{\mathrm{it}}$ as a ratio, we control for a household’s overall location within the village milieu so that living on the outskirts (or in the centre) of a village does not have a disproportionate effect on one’s access to improved seed (technology transfer). Finally, by lagging the variables we resolve the potential simultaneity of access problem in which we cannot distinguish who (farmer or neighbour) first had access to improved seeds (technology transfer).^6^

To instrument for access to technology transfer and improved seed we use a control function (CF) approach developed by Smith and Blundell (1986). Our choice of the CF approach, instead of the standard two-stage least squares (2SLS) approach is driven by the prevalence of zeros in our adoption equation, giving it the properties of a non-linear corner solution. While in linear models CF leads to the 2SLS estimator, in non-linear models these two approaches will give different results (Imbens and Wooldridge, 2007, Lewbel, 2004). In these cases, the CF approach is more efficient then standard 2SLS.^7^

This involves first estimating the reduced form probit model to predict the access to technology transfer and improved seed (Wooldridge, 2010). We then calculate the generalized residuals and include them in the structural model of improved chickpea adoption specified in Eq. (3). In the reduced form equation we include all exogenous variables from the structural model, year and village dummies, as well as the means of time-varying variables to control for unobserved heterogeneity.

#### 5.1.3 Censored dependent variable

A final estimation issue in the adoption equation is how to deal with the censored dependent variable. As mentioned previously, between 21 and 68 percent of households are non-adopters in any given year. The prevalence of households that grow no improved chickpea means that our dependent variable is censored and our model is more appropriately expressed as a non-linear corner solution.

(6) $$K_{\mathrm{it}}=\max(0\text{,}\alpha +\beta_{1}X_{\mathrm{it}}^{\mathrm{TT}}+\beta_{2}X_{\mathrm{it}}^{\mathrm{IS}}+T_{\mathrm{it}}\theta +Z_{i}\zeta +D_{t}+v+{∊}_{\mathrm{it}})$$

This specification allows for the decision not to adopt improved chickpea to be optimal for some farming households. In this situation the Tobit estimator may be used since zeros represent household choice and not missing data due to incidental truncation. However, the Tobit estimator implies that the decision to adopt and the degree of adoption are determined by the same process. We follow Ricker-Gilbert et al., 2011, Bezu et al., 2014 in using a double hurdle model to estimate adoption. The double hurdle model, as developed by Cragg (1971) relaxes the restrictions of the Tobit estimator. The decision to adopt, the first hurdle, is estimated using a probit. Then the degree or intensity of adoption, the second hurdle, is estimated using a truncated normal regression model. In each hurdle we include all exogenous variables, our endogenous variables, the generalized residuals, the means of time-varying variables and year and village dummies. Since we include the generalized residuals from the reduced form equation we bootstrap the standard errors, since they are likely to be biased.

### 5.2 Estimating the impact of improved chickpea adoption

While an important metric, estimating chickpea adoption is not our primary focus. Rather, we are interested in understanding the welfare impacts for those who adopt improved chickpea. To do this, we specify Eq. (2) in our conceptual model as the following

(7) $$Y_{\mathrm{it}}=\alpha_{i}+\phi K_{\mathrm{it}}+T_{\mathrm{it}}\theta +D_{t}+{∊}_{\mathrm{it}}$$

where $Y_{\mathrm{it}}$ is our welfare measure variously defined as total net income, net income per capita, an indicator for household poverty status at 1.25 USD PPP and at 2.00 USD PPP. Other variables are as previously defined. As with our model of improved chickpea adoption, our model of household welfare suffers from two potential sources of endogeneity. The first potential source of endogeneity comes from unobserved heterogeneity. Time-invariant household characteristics which are unobserved may be correlated both with adoption and with our welfare measures. Here again we have the issue of selection bias, where some households, depending on skill, risk preferences, etc., are likely to adopt a new technology while also having higher welfare measures *ex ante*. Given that our specification of household welfare is linear, we no longer have the incidental variables problem and utilize fixed effects to control for unobservables.

The second potential source of endogeneity comes from unobserved shocks that jointly influence the decision to adopt improved chickpea as well as a household’s welfare status. Such shocks could be covariate (such as weather events) or idiosyncratic (such as a death in the family). We include mean rainfall over the last five years and its standard deviation to help control for covariate shocks related to weather.^8^ To control for additional, primarily idiosyncratic, shocks we follow Bezu et al. (2014) in using the unconditional expected values of area planted with improved chickpea as an instrument for observed adoption. First, we estimate adoption using the double hurdle model as previously outlined. Second, we calculate the unconditional expected values of adoption using the predicted values from the double hurdle model. Finally, we estimate the welfare equation using fixed effects and instrumenting for our variable of interest (observed area of land under improved chickpea) with the expected values of adoption.^9^ In general this approach is more efficient than standard 2SLS and it is also more efficient than the CF approach in linear models (Wooldridge, 2003).

The variables which are excluded from the outcome equation and therefore provide us with the exogenous variation necessary for identification are: soil characteristics, distance to market, access to seed and access to technology transfer. While we do not expect that soil characteristics and distance to market will be directly correlated with income after controlling for improved chickpea planted, they also do not provide enough variation to identify the instrument for both years separately (on their own they could only identify a single value for each household, not a value for each household in each year). Therefore, we rely on access to seed and technology transfer, variables that also satisfy the exclusion restriction, to provide variation in our instrument over time.

## 6 Results

We first estimate Eq. (6) using the correlated random effects double hurdle model treating access to improved seed and technology transfer as exogenous. In this specification both terms are significant and positively correlated with the probability of planting improved chickpea. However, neither are significant in the second hurdle (see columns (1) and (2) in Table 4). Next we treat access to improved seed and technology transfer as endogenous and instrument for these terms using each of our distance weighted measures of access by including the generalized residuals from each of the first stage, reduced form regressions.^10^ The coefficient for the generalized residual for access to improved seed is significant in the first hurdle (see column (3) in Table 4), suggesting that access to improved seed is endogenous to the decision to adopt improved chickpea. The coefficients for participation in technology transfer and its generalized residual are not significant in the first hurdle, but are significant in the second hurdle (see column (4) in Table 4), which indicates that participation in technology transfer may not be important in the decision to adopt but is important in the extent of adoption.

Examining the other variables in the double hurdle model, the extent of adoption, but not adoption, is strongly and positively correlated with landholding. This result indicates that while additional landholding may or may not influence adoption, households with more land allocate larger tracts to improved varieties. Wealthier households were both more likely to adopt and allocated more land to improved chickpea, which confirms our descriptive finding that adopters are wealthier and that richer households may have been targeted by extension. Off-farm income is negatively related to chickpea adoption, suggesting that having additional sources of income reduces a household’s ability or interest in adopting new agricultural technologies. Age and education of the head of household do not influence the choice to adopt but older and less educated household heads allocate less land to improved varieties, possibly indicating risk-aversion and technology mistrust as suggested by Bezu et al. (2014).

The fixed effects models provide evidence on the relationship between improved chickpea adoption and our various welfare indicators (Table 5). The model is robust to our specification of income, showing a positive impact on both income per capita (Column (1)) and household income (Column (2)). Controlling for all other factors, a 10 percent increase in the area planted with improved chickpea is associated with a 12.6 percent increase in income per capita and a 12.3 percent increase in total income. Considering the impact on poverty, the fixed effects linear probability model indicates that adopting improved chickpea varieties can reduce the probability of a household being below the $2.00 poverty line but is unable to reduce the probability of a household being below the $1.25 poverty line. A 10 percent increase in the area planted with improved chickpea reduces the probability of being below the median poverty line by 3.9 percent. Changes in other covariates have the expected signs where they are significant.^11^ We conclude that adoption of improved chickpea increases household income and that adoption can increase income to such a degree that it can raise household above the median poverty line. But, this increase in income is insufficient to raise the poorest households out of poverty.

In order to verify the validity of our results to changes in our specification we conduct a variety of robustness checks (Table 6). In row (1) we present, for purposes of comparison, our primary estimation results. In row (2) we present results using the predicted values from the double hurdle model where access to improved seed and technology transfer are treated as exogenous. In row (3) we present results using a trimmed data set, where the top and bottom one percent of households, based on income per capita for the 2006/07 season, are removed. In row (4) we present results similar to our primary results but include village-time trends at all levels instead of just village indicators. In row (5) we replace our preferred measure of the extent of adoption (area planted) with the amount of seed planted. In row (6) we replace the control function in our model of adoption with a two-stage instrumented Tobit prior to our fixed effects regression. Across all these alternative specifications we find that improved chickpea adoption has a strong positive impact on household income as well as a consistently negative impact on the probability of being below the median poverty line. However, our initial finding that improved chickpea adoption has no effect on households below the $1.25 poverty line turns out to lack robustness. In some specifications we find a significant impact of improved chickpea adoption in reducing poverty while in other specification we find no impact at all.

While our econometric results provide strong evidence that adoption of improved chickpea varieties increases income and reduces median level poverty, Dercon and Christiaensen (2011), point out that households may not be equally able to capitalize on new technology. The very poorest households may have a reduced capacity to cope with shocks, due to a lack of capital, knowledge or access to markets, which keeps them caught in a poverty trap. Conversely, the wealthiest households may no longer be as reliant on agriculture and therefore may be less impacted by a new agricultural technology. In order to explore these possible heterogeneous effects of adoption we allow our coefficient of interest to vary based on a household’s initial land ownership. We re-specify Eq. (7) as

(8) $$Y_{\mathrm{it}}=\alpha_{i}+\sum \phi_{q}(Q_{q}K_{\mathrm{qit}})+T_{\mathrm{it}}\theta +D_{t}\delta +{∊}_{\mathrm{it}}$$

where Q is an indicator for the land ownership quartile (indexed by q=1,…,4) to which household i belongs. By allowing $\phi_{q}$ to vary by landholding we can test if adoption has heterogeneous effects on changes in welfare across initial wealth status.^12^ Results presented in Table 7 show that the impact of adoption on welfare is strongly significant and positive for households in the three lower quartiles. However, adoption did not have a significant effect on welfare for the largest landholding households.

## 7 Discussion

Our results show the dramatic increase in improved chickpea adoption has had a strong positive effect on household welfare. This confirms the findings by Asfaw et al. (2012) who found a similar positive effect of improved chickpea adoption using the first round of data collected. There are several potential channels through which improved chickpea can increase household income. While a full exploration of these channels is beyond the scope of the current paper, we do provide some descriptive evidence on this issue. Table 8 presents inputs used in chickpea production as well as yield and sales information by adoption type. For inputs, improved varieties require costlier seed, use slightly more fertilizer and require more chemicals. Given that adopters of improved varieties plant significantly more land to chickpea, the cultivation of improved varieties also requires more labour. Households meet this increased labour demand by hiring more workers while family labour remains constant.

The increase in input use associated with improved chickpea cultivation contributes to significantly higher yields. These increased yields allow households to sell a larger share of their production into the market. While improved varieties command only a small mark-up, the return to improved chickpea is significantly higher given the significantly larger volume of sales. All this leads to chickpea sales making up a larger share of total income for those who adopt improved varieties. Our findings provide evidence that the adoption of improved chickpea can contribute to household income and poverty reduction in rural Ethiopia.

While we find strong impacts on income and evidence that adoption of improved chickpea can reduce median level poverty, we find little evidence that adoption was able to lift the poorest households above the $1.25 poverty line. One explanation for this result comes from Abro et al. (2014) who found that the poorest households in Ethiopia are more prone to income shocks. A particularly strong shock during our study period was the double digit inflation in Ethiopia (World Bank, 2015a). Dercon and Christiaensen (2011) suggested that the poorest households have a reduced capacity to cope with such large shocks while Dercon et al. (2012) found evidence of a serious “growth handicap” for poor households in Ethiopia, which contributes to poverty persistence by inducing permanently lower outcomes. This suggests that additional efforts, beyond adoption of improved chickpea, may be required to lift the poorest households out of poverty.

We also fail to find evidence that improved chickpea adoption had a significant impact on the income of the wealthiest households, in terms of landholding. We hypothesize that this is due to households with large landholding being more diversified in their income sources. Households in the top quartile based on initial landholding were more likely to adopt improved chickpea: 68 percent of these households adopted compared to 55 percent of households in the lower three quartiles (significantly different at the 99 percent level). These households also planted a significantly larger area to improved chickpea (again significant at the 99 percent level). However, large landholding households were no different than households in the lower three quartiles when we examine the share of land area allocated to improved chickpea. Moreover, improved chickpea made up a smaller share of income for large landholding households compared to households in the lower three quartiles (significant at the 99 percent level). We interpret this to mean that while large landowning households adopted improved chickpea, the extent to which they reallocated land to chickpea was not large enough to make a significant impact on their income. Further research is needed to identify the mechanisms that can explain the disparate effects of adoption.

It has been suggested that improved chickpea varieties present an environmentally friendly technology for poverty reduction in Ethiopia (Kassie et al., 2009). This is important as increasing agricultural productivity by sustainably intensifying output per unit of land is deemed essential in Ethiopia (Josephson et al., 2014). Smallholder farming in Ethiopia is often subject to environmental disturbances such as drought, untimely or uneven distribution of rainfall and incidences of pests and diseases (Teklewold et al., 2013). Improved chickpea varieties are disease-resistant and drought tolerant. Moreover, chickpeas fix atmospheric nitrogen in soils, allowing farmers to save on chemical fertilizer use in subsequent cereal crops (Giller, 2001). As indicated by Lee (2005), environmentally sustainable technologies need to simultaneously generate positive agronomic and economic benefits if they are to achieve wide adoption. Our analysis provides evidence of the positive effect of chickpea adoption on both income and poverty reduction. Given the economic importance of chickpea in Ethiopia and the beneficial biotic and nutritional characteristics of legumes, improved chickpea seem to be a promising technology for sustainable intensification in Ethiopia.

Understanding the effects of improved chickpea adoption on household welfare is an important step in developing policies for chickpea growing areas in Ethiopia. Average adoption rates in Ethiopia are estimated to be much lower than those observed in our study area, though country-wide adoption figures are not available. In order to assess the potential for further upscaling it would be helpful to analyse the processes that facilitated the dramatic increase in adoption in our study area. Policies that remove obstacles for the diffusion of improved chickpea varieties can be important for addressing smallholder welfare. For instance, seed replenishment rates are low and attention is therefore needed to ensure that there is a sufficient and consistent supply of affordable quality chickpea seed. It is unlikely that the private sector will take up this challenge because farmers can re-use seed for up to five seasons (Jones et al., 2006). Hence, support is needed to strengthen farmer based seed systems to ensure improve accessibility of improved chickpea varieties. Ultimately, our results suggest that improved chickpea varieties could very well be an attractive pathway for rural development in Ethiopia.

## 8 Conclusions

This article answers the empirical question: what is the impact of improved chickpea adoption on household welfare in rural Ethiopia? We estimate chickpea adoption using a double hurdle model with correlated random effects and then use predicted chickpea area from the double hurdle model to instrument for adoption in the fixed effects welfare estimations. We find that improved chickpea adoption significantly increases household income while also reducing median level poverty. To explore the possibility of heterogeneous effects of adoption, we disaggregate results by initial landholding and find that adoption favoured all but the largest landholding households, who adopted but not to an extent where adoption significantly affected their large and diverse income streams. Because our data comes from a region suitable for chickpea production, our positive findings are an upper bound on the potential for sustainable intensification of chickpea production in Ethiopia. With this caveat, our results provide concrete evidence for policies targeting poverty reduction in rural Ethiopia.

## Figures and Tables

**Table A:** Correlated random effects probit model of access to technology transfer and seed.

	(1)	(2)
	Access technology transfer (yes = 1)	Buys improved seed (yes = 1)
*Lagged weighted distance to tech. transfer*	−1.488^∗∗∗^	
	(0.542)	
*Lagged weighted distance to seed*		−0.891^∗∗∗^
		(0.267)
Age head (yrs)	−0.015^∗∗∗^	−0.009^∗∗^
	(0.005)	(0.004)
Education head (yrs)	−0.006	0.016
	(0.019)	(0.018)
Male head (yes = 1)	−1.092^∗∗^	0.395
	(0.437)	(0.447)
Household size (No.)	−0.144^∗∗∗^	−0.012
	(0.043)	(0.046)
Dependents (%)	0.007^∗^	−0.002
	(0.004)	(0.004)
Off-farm income (yes = 1)	−0.149	−0.090
	(0.152)	(0.134)
Ln initial asset ownership (USD PPP)	0.096^∗^	0.016
	(0.057)	(0.052)
Ln land owned (ha)	0.451^∗^	−0.043
	(0.237)	(0.188)
Ln market distance (min)	−0.015	0.228
	(0.294)	(0.238)
Average rainfall (mm)	0.013	0.006
	(0.010)	(0.011)
Standard deviation rainfall (mm)	0.006	0.005
	(0.010)	(0.009)
Black soil (yes = 1)	−0.196	−0.336^∗^
	(0.238)	(0.204)
Sandy soil (yes = 1)	0.056	0.086
	(0.128)	(0.106)
Mixed soil (yes = 1)	0.032	0.097
	(0.117)	(0.098)

Observations	1212	1212
Households	606	606

*Note*: Column (1) presents first stage regression results predicting access to technology transfer with *lagged weighted distance to technology transfer* as an instrument. Column (2) presents first stage regression results predicting access to improved seed with *lagged weighted distance to seed* as an instrument. Fully robust standard errors in parentheses (^*^p < 0.10, ^**^p < 0.05, ^***^p < 0.01). Regressions include the means of time-variant variables, year dummies and village dummies.

**Table 1:** Descriptive statistics improved chickpea adoption.

	2006/07		2009/10		2013/14	
	Mean	s.d.	Mean	s.d.	Mean	s.d.
*Panel A: Balanced sample*						
Chickpea (yes = 1)	0.655	0.476	0.805	0.396	0.881	0.324
Improved variety (1 = yes)	0.312	0.464	0.630	0.483	0.790	0.407
Improved chickpea area (ha)	0.172	0.390	0.327	0.414	0.425	0.427
Improved chickpea seed (kg)	34.23	79.27	60.70	80.05	89.60	101.8
Improved chickpea share area (%)	5.925	11.67	12.11	12.96	18.90	14.24
Improved chickpea share income (%)	7.023	13.80	15.84	16.94	16.23	13.21

Observations	606		606		606	

*Panel B: Chickpea growers*						
Improved variety (1 = yes)	0.476	0.500	0.783	0.413	0.897	0.304
Improved chickpea area (ha)	0.263	0.457	0.406	0.425	0.482	0.423
Improved chickpea seed (kg)	52.25	93.04	75.38	82.78	101.7	102.7
Improved chickpea share area (%)	9.044	13.41	15.03	12.82	21.45	13.24
Improved chickpea share income (%)	10.72	15.85	19.68	16.77	18.42	12.56

Observations	397		488		534	

*Note*: Panel A displays means and standard deviations of improved chickpea adoption indicators by year for the balanced sample. Panel B displays means and standard deviations of improved chickpea adoption indicators by year for households that grow chickpeas.

**Table 2:** Socio-economic characteristics of adopters and non-adopters.

	2006/07		*t*-test	2009/10		*t*-test	2013/14		*t*-test
	Non-adopter	Adopter		Non-adopter	Adopter		Non-adopter	Adopter	
*Demographics*									
Household size (no.)	6.08	6.76	^⁎⁎⁎^	6.00	6.59	^⁎⁎⁎^	5.63	5.81	
Dependents (%)	42.9	45.4		39.0	40.9		39.9	34.9	^⁎⁎^
Male head (yes = 1)	0.93	0.96		0.94	0.95		0.91	0.91	
Education head (years)	1.59	1.98	^⁎^	1.87	1.99		2.14	1.8	
Age head (years)	46.3	47.9		49.3	48.1		50.3	52.0	

*Welfare*									
Total net income (USD)	4541	7760	^⁎⁎⁎^	4145	7008	^⁎⁎⁎^	3404	4696	^⁎⁎⁎^
Income per capita (USD)	837	1232	^⁎⁎⁎^	806	1175	^⁎⁎⁎^	670	885	^⁎⁎⁎^
Land owned (ha)	2.01	2.67	^⁎⁎⁎^	2.00	2.41	^⁎⁎⁎^	1.94	2.16	^⁎^
Value assets (USD)	363	477	^⁎⁎^	325	376	^⁎^	493	722	^⁎⁎⁎^
Poor household (<$1.25)	0.28	0.11	^⁎⁎⁎^	0.37	0.20	^⁎⁎⁎^	0.48	0.27	^⁎⁎⁎^
Poor household (<$2.00)	0.57	0.32	^⁎⁎⁎^	0.58	0.39	^⁎⁎⁎^	0.70	0.54	^⁎⁎⁎^

Observations	417	189		224	382		127	479	

Significance of *t*-tests are reported as ^⁎^p < 0.10, ^⁎⁎^p < 0.05, ^⁎⁎⁎^p < 0.01.

**Table 3:** Descriptive statistics for variables used in the econometric analysis.

	2006/07		2009/10		2013/14	
	Mean	s.d.	Mean	s.d.	Mean	s.d.
Distance to neighbours (km)	94.54	173.0	94.54	173.0	94.54	173.0
Technology transfer (1 = yes)	0.013	0.114	0.127	0.333	0.150	0.358
Distance to technology transfer (km)	1.079	4.390	8.673	23.50	12.05	28.39
Lag weighted dist. tech. transfer (IV)	–	–	0.013	0.039	0.124	0.151
Access to improved seed (yes = 1)	0.195	0.396	0.195	0.396	0.186	0.390
Distance to improved seed (km)	17.32	36.46	18.47	42.10	21.54	48.15
Lag weighted distance seed (IV)	–	–	0.191	0.221	0.201	0.184
Age head (years)	46.81	12.08	46.81	12.08	46.81	12.08
Education head (years)	1.713	2.647	1.713	2.647	1.713	2.647
Male head (1 = yes)	0.936	0.246	0.942	0.233	0.914	0.280
Household size (no.)	6.295	2.250	6.368	2.358	5.772	2.089
Dependents (%)	43.70	20.49	40.21	19.62	35.98	21.60
Off-farm income (1 = yes)	0.276	0.447	0.246	0.431	0.282	0.450
Land owned (ha)	2.215	1.308	2.257	1.299	2.122	1.281
Initial asset ownership (USD)	398.4	560.7	398.4	560.7	398.4	560.7
Walking distance to market (min)	196.5	84.50	196.5	84.50	196.5	84.50
Average rainfall past 5 seasons (mm)	598.0	47.65	622.4	52.93	599.2	50.91
St. dev. rainfall past 5 seasons (mm)	97.70	15.50	57.85	12.64	81.18	12.04
Black soil (yes = 1)	0.969	0.174	0.969	0.174	0.969	0.174
Sandy soil (yes = 1)	0.777	0.416	0.777	0.416	0.777	0.416
Mixed soil (yes = 1)	0.246	0.431	0.246	0.431	0.246	0.431

Observations	606		606		606	

**Table 4:** Adoption decision: Cragg’s double hurdle model using correlated random effects estimation.

	Technology transfer and seed access exogenous		Technology transfer and seed access endogenous	
	(1) Probability of planting (Hurdle 1)	(2) Area planted (Hurdle 2)	(3) Probability of planting (Hurdle 1)	(4) Area planted (Hurdle 2)
Access to technology transfer	6.370^∗∗∗^	0.051	−0.362	0.436^∗∗∗^
(yes = 1)	(1.337)	(0.032)	(1.179)	(0.163)
Generalized residual access	–	–	0.608	−0.233^∗∗^
Technology transfer			(0.650)	(0.092)
Access to improved	0.663^∗∗^	0.003	1.608	0.181
Seed (yes = 1)	(0.265)	(0.038)	(2.013)	(0.194)
Generalized residual access seed	–	–	3.026^∗∗∗^	−0.074
			(1.013)	(0.111)
Age head (yrs)	−0.006	−0.002^∗^	−0.020^∗∗∗^	−0.001
	(0.006)	(0.001)	(0.007)	(0.002)
Education head (yrs)	0.002	0.014^∗∗^	0.023	0.014^∗∗^
	(0.028)	(0.007)	(0.029)	(0.007)
Male head (yes = 1)	0.208	−0.053	0.507	−0.038
	(0.693)	(0.096)	(0.609)	(0.111)
Household size (no.)	−0.084	0.026^∗∗∗^	−0.114^∗^	0.043^∗∗∗^
	(0.063)	(0.010)	(0.066)	(0.011)
Dependents (%)	0.000	−0.001	0.002	−0.001
	(0.005)	(0.001)	(0.005)	(0.001)
Off-farm income (yes = 1)	−0.392^∗∗^	0.015	−0.495^∗∗∗^	0.002
	(0.190)	(0.039)	(0.174)	(0.036)
Ln asset ownership (USD)	0.117^∗^	0.088^∗∗∗^	0.151^∗∗^	0.083^∗∗∗^
	(0.069)	(0.016)	(0.074)	(0.016)
Ln land owned (ha)	0.247	0.263^∗∗∗^	0.332	0.257^∗∗∗^
	(0.306)	(0.055)	(0.280)	(0.054)
Ln distance to market (km)	−0.420	0.052	−0.243	0.053
	(0.368)	(0.070)	(0.395)	(0.070)
Average rainfall (mm)	0.032^∗∗^	0.004	0.029^∗∗^	0.002
	(0.013)	(0.003)	(0.013)	(0.003)
St. dev. of rainfall (mm)	0.057^∗∗∗^	0.001	0.051^∗∗∗^	−0.002
	(0.010)	(0.003)	(0.010)	(0.003)
Black soil (yes = 1)	−0.137	0.020	−0.633^∗^	0.059
	(0.353)	(0.073)	(0.377)	(0.074)
Sandy soil (yes = 1)	−0.029	−0.000	0.095	−0.006
	(0.143)	(0.034)	(0.146)	(0.035)
Mixed soil (yes = 1)	0.151	−0.025	0.261^∗^	−0.025
	(0.125)	(0.035)	(0.138)	(0.033)
Sigma		0.281^∗∗∗^		0.279^∗∗∗^
		(0.012)		(0.012)

Observations	1212		1212	
Households	606		606	
Bootstrapping replications	1000		1000	

*Note*: The first double hurdle regression (column 1 and 2) treats technology transfer and access to seed as exogenous to the decision to adopt. The second double hurdle regression (column 3 and 4) includes first stage residuals to control for potential endogeneity of technology transfer and access to seed. Results from the first stage reduced form regression are presented in Appendix Table A. Fully robust bootstrapped standard errors in parentheses (^*^p < 0.10, ^**^p < 0.05, ^***^p < 0.01). Regressions include the means of time-variant variables, year dummies and village dummies.

**Table 5:** Adoption impact on income and poverty: fixed effects instrumental variable estimation.

	(1)	(2)	(3)	(4)
	Ln income per capita	Ln household income	Poor (<$1.25)	Poor (<$2.00)
Ln improved chickpea area (ha)	1.261^∗∗^	1.226^∗∗^	−0.274	−0.388^∗^
	(0.551)	(0.605)	(0.203)	(0.207)
Male head (yes = 1)	0.177	0.189	−0.196^∗∗^	0.056
	(0.185)	(0.187)	(0.098)	(0.112)
Household size (No.)	−0.113^∗∗^	0.058	0.064^∗∗∗^	0.087^∗∗∗^
	(0.045)	(0.051)	(0.012)	(0.013)
Dependents (%)	−0.004	−0.004	0.000	0.001
	(0.004)	(0.004)	(0.001)	(0.001)
Off-farm income (yes = 1)	0.208^∗∗∗^	0.211^∗∗∗^	−0.069^∗^	−0.067
	(0.068)	(0.069)	(0.038)	(0.042)
Ln land owned (ha)	−0.019	−0.079	−0.293^∗∗∗^	−0.241^∗∗∗^
	(0.285)	(0.328)	(0.066)	(0.070)
Average rainfall (mm)	0.001	0.004	0.003	0.005
	(0.008)	(0.009)	(0.003)	(0.003)
St. dev. rainfall (mm)	0.021^∗^	0.024^∗^	−0.006^∗∗^	−0.006^∗∗^
	(0.011)	(0.013)	(0.003)	(0.003)

Kleibergen-Paap Wald F-statistic	67.176^∗∗^	67.176^∗∗^	67.176^∗∗^	67.176^∗∗^
Observations	1212	1212	1212	1212
Households	606	606	606	606
Bootstrapping replications	1000	1000	1000	1000

*Note*: Columns present fixed effects instrumental variables regressions for four different measures of household welfare as the dependent variable. In all models Ln improved chickpea area is treated as endogenous and instrumented with the predicted improved chickpea area from the endogenous double hurdle model in column (4) of Table 4. Fully robust bootstrapped standard errors in parentheses (^*^p < 0.10, ^**^p < 0.05, ^***^p < 0.01). In addition to household fixed effects, regressions include year dummies.

**Table 6:** Robustness checks of adoption impact.

	(1)	(2)	(3)	(4)
	Ln income per capita	Ln household income	Poor (<$1.25)	Poor (<$2.00)
(1) Primary results	1.261^∗∗^	1.226^∗∗^	−0.274	−0.388^∗^
	(0.551)	(0.605)	(0.203)	(0.207)
(2) Tech. transfer and seed access exogenous	1.327^∗∗^	1.328^∗^	−0.293	−0.382^∗^
	(0.632)	(0.703)	(0.206)	(0.201)
(3) 1% trim	1.341^∗∗^	1.297^∗∗^	−0.335^∗^	−0.420^∗∗^
	(0.564)	(0.616)	(0.199)	(0.209)
(4) Village time interactions	1.305^∗∗^	1.293^∗∗^	−0.361^∗^	−0.440^∗∗^
	(0.568)	(0.620)	(0.215)	(0.211)
(5) Ln improved chickpea seed (kg)	0.073^∗^	0.069^∗^	−0.027	−0.038^∗∗^
	(0.039)	(0.042)	(0.018)	(0.019)
(6) Two-stage Tobit	2.088^∗∗∗^	2.077^∗∗∗^	−0.603^∗^	−0.456
	(0.742)	(0.799)	(0.333)	(0.338)

Observations	1212	1212	1212	1212
Number of households	606	606	606	606

*Note*: Columns present fixed effects instrumental variables regressions for four different measures of household welfare as the dependent variable. Row (1) reports, for purposes of comparison, the results found in Table 5. Row (2) reports results using the predicted improved chickpea area from the exogenous double hurdle model in column (2) of Table 4 as an instrument for observed values. Row (3) reports results from the balanced panel when we trim the top and bottom 1% of observations based on initial income per capita. Row (4) includes village specific time trends to control for village specific trends that may be correlated with chickpea adoption. Row (5) presents an alternative specification in which the extent of adoption is measured by the quantity of improved chickpea seeds planted. Row (6) reports results in which we replace the CF double hurdle with a more standard two-stage instrumented Tobit prior to our fixed effects regression. Fully robust bootstrapped standard errors in parentheses (^*^p < 0.10, ^**^p < 0.05, ^***^p < 0.01). Regressions include explanatory variables from Table 5, household fixed effects and year dummies.

**Table 7:** Fixed effects estimation: adoption impact by initial land ownership.

	(1)	(2)
	Ln income per capita	Ln household income
Quartile 1 ∗ Ln improved chickpea area	2.227^∗∗∗^	2.424^∗∗∗^
	(0.752)	(0.821)
Quartile 2 ∗ Ln improved chickpea area	1.269^∗^	1.335^∗^
	(0.732)	(0.809)
Quartile 3 ∗ Ln improved chickpea area	1.469^∗∗^	1.315^∗^
	(0.677)	(0.738)
Quartile 4 ∗ Ln improved chickpea area	0.180	0.109
	(1.193)	(1.306)

Observations	1212	1212
Number of households	606	606

*Note*: Columns present fixed effects instrumental variables regression results similar to those presented in Columns (1) and (2) of Table 5 except that the instrumented variable is interacted with an indicator for the initial land quartile to which each household belongs. Fully robust bootstrapped standard errors in parentheses (^*^p < 0.10, ^**^p < 0.05, ^***^p < 0.01). Regressions include explanatory variables from Table 5, household fixed effects and year dummies.

**Table 8:** Costs and benefits of chickpea production.

	Full sample		*t*-test
	Non-adopter	Adopter	
*Costs*			
Chickpea area (ha)	0.19	0.65	^⁎⁎⁎^
Chickpea seed (USD/ha)	183	261	^⁎⁎⁎^
Chickpea fertilizer (USD/ha)	11.4	19.2	^⁎⁎^
Chickpea chemical (USD/ha)	19.3	41.7	^⁎⁎⁎^
Chickpea hired labour (USD/ha)	24.2	46.1	^⁎⁎⁎^
Chickpea family labour (days/ha)	78.3	74.3	

*Benefits*			
Chickpea yields (kg/ha)	1875	2338	^⁎⁎⁎^
Sold chickpeas (yes = 1)	0.37	0.87	^⁎⁎⁎^
Share of chickpea production sold (%)	54.3	58.0	^⁎^
Chickpea sales (kg)	401	857	^⁎⁎⁎^
Chickpea sales price (USD/kg)	1.25	1.33	^⁎⁎^
Net returns to chickpea sales (USD)	739	1727	^⁎⁎⁎^
Chickpea sales as share of income (%)	21.6	38.6	^⁎⁎⁎^

Observations	369	1050	

Significance of *t*-tests are reported as ^⁎^p < 0.10; ^⁎⁎^p < 0.05; ^⁎⁎⁎^p < 0.01.

## Footnotes

1 Access to improved seed includes households that bought (from the market), borrowed (from a revolving seed fund) or were given (by friend/family/neighbour) improved seed. Technology transfer activities include farm trials or demonstrations, farmer field days, farmer field schools and seminars.

2 Tropical Legumes II was a Bill and Melinda Gates Foundation project to enhance grain legume productivity and production to increase poor farmers’ income in drought-prone areas of Sub-Saharan Africa and South Asia. It was led by ICRISAT in partnership with CIAT, IITA and NARS partners and has just been renewed for a third phase (Tropical Legumes III). For more information see http://www.icrisat.org/TropicalLegumesII/

3 For further details we refer to Asfaw et al. (2012), who describe the sampling for the first round of the panel dataset and provide a more detailed account of the sampling strategy.

4 Results are available from the authors upon request.

5 See Deaton (2010) for a thorough discussion of the measurement of poverty and the role of PPP price indexes.

6 We thank an anonymous reviewer for pointing out this potential confounder in the non-lagged version of our instruments, though, it still does not completely control for all potential unobservables. Having controlled for time-invariant household and village level effects and (in a robustness check) for time-variant village level effects, what remains are time-variant shocks at the sub-village level. These shocks would need to systematically only effect the ability to access seeds or technology transfer for a portion of residents in a village. If these events were then also correlated with a given household’s decision to adopt chickpea in the following time period, we would have failed to fully identify the adoption decision. Our results should be interpreted with this in mind.

7 Note that this efficiency comes at the cost of additional assumptions. These assumptions were originally laid out in Rivers and Vuong (1988) and relaxed in Wooldridge (2005) and are more restrictive than standard assumptions required in 2SLS estimation.

8 Our rainfall data comes from the Climate Hazards Group InfraRed Precipitation with Station data (CHIRPS). See (Funk et al., 2015) for detailed information on the data.

9 We use a linear probability model to estimate the poverty regressions instead of a probit or logit as these non-linear models carry several costs as outlined by Dercon et al. (2009).

10 See Appendix A for results from the first stage regressions of access to improved seed and technology transfer.

11 Running the model with value of assets and tropical livestock units owned as dependent variables did not give significant results. We hypothesize that there was insufficient time for adoption to contribute to asset accumulation.

12 This method is similar to that used by Hurst et al., 2010, Michler and Balagtas, 2016 to test for heterogeneous effects across groups without splitting the random sample based on a non-random criteria.
